# The 4q12 Amplicon in Malignant Peripheral Nerve Sheath Tumors: Consequences on Gene Expression and Implications for Sunitinib Treatment

**DOI:** 10.1371/journal.pone.0011858

**Published:** 2010-07-29

**Authors:** Jan Zietsch, Nicolas Ziegenhagen, Frank L. Heppner, David Reuss, Andreas von Deimling, Nikola Holtkamp

**Affiliations:** 1 Department of Neuropathology, Charité - Universitätsmedizin Berlin, Berlin, Germany; 2 Department of Neuropathology, Ruprecht-Karls-University Heidelberg, and Deutsches Krebsforschungszentrum Heidelberg, Heidelberg, Germany; Institute of Cancer Research, United Kingdom

## Abstract

**Background:**

Malignant peripheral nerve sheath tumors (MPNST) are highly aggressive tumors which originate from Schwann cells and develop in about 10% of neurofibromatosis type 1 (NF1) patients. The five year survival rate is poor and more effective therapies are needed. Sunitinib is a drug targeting receptor tyrosine kinases (RTK) like PDGFRα, c-Kit and VEGFR-2. These genes are structurally related and cluster on chromosomal segment 4q12.

**Methodology/Principal Findings:**

Here we characterize this region by multiplex ligation-dependent probe amplification (MLPA) in MPNST. Our probe set encompasses the 3 adjacent RTK genes (*PDGFRA*, *KIT*, *KDR*) and 6 flanking genes. We found amplification of several genes within this region in a subset of MPNST and MPNST cell lines. Transcript and protein expression of *PDGFRA* matched well with its increased copy number suggesting a central role of *PDGFRA* within the amplicon. Studying the effect of sunitinib on 5 MPNST cell lines revealed that cell line S462 harboring the 4q12 amplicon was extremely sensitive to the drug with an IC_50_ below 1.0µM. Moreover, sunitinib induced apoptosis and prevented PDGF-AA induced signaling via PDGFRα as determined by western blotting. Co-expression of VEGF and its receptor VEGFR-2 (*KDR*) was present in MPNST cell lines suggesting an autocrine loop. We show that VEGF triggered signal transduction via the MAPK pathway, which could be blocked by sunitinib.

**Conclusions/Significance:**

Since multiple receptors targeted by sunitinib are expressed or over-expressed by MPNST cells sunitinib appears as an attractive drug for treatment of MPNST patients. Presence of the 4q12 amplicon and subsequent over-expression of *PDGFRA* might serve as predictive markers for efficacy of sunitinib.

## Introduction

Neurofibromatosis type 1 (NF1) is a genetic disorder that affects about one in 3000 individuals and manifests with a broad range of clinical symptoms [1]. Neurofibromas are a hallmark of the disease and develop in virtually all NF1 patients. Plexiform neurofibromas (pNF) can undergo malignant progression and form malignant peripheral nerve sheath tumors (MPNST). In contrast, dermal neurofibromas (dNF) show no tendency towards malignant transformation. For the time being, surgical intervention is the most effective treatment for patients with MPNST. However, due to the invasive growth pattern complete tumor removal is frequently not possible. MPNST respond only poorly to conventional chemo- and radiotherapies and effective alternative therapies are not available yet. In order to develop novel strategies for target directed therapy a better knowledge on molecules contributing to malignant progression is needed.

Recently, we demonstrated gene amplification of adjacent genes encoding platelet-derived growth factor receptor alpha (PDGFRα) and c-Kit in a subset (19%) of MPNST [2]. To determine whether more genes are amplified on chromosomal segment 4q12 we analyzed 9 genes distributed over 5 Mb by multiplex ligation-dependent probe amplification (MLPA). A similar study was performed recently with gliomas [3]. The examined region contains a cluster of 3 adjacent receptor tyrosine kinase (RTK) genes (*PDGFRA*, *KIT* and *KDR*). The corresponding proteins, namely PDGFRα, c-Kit and vascular endothelial growth factor 2 (VEGFR-2), play a central role in cancer biology and have been reported with genetic alterations and strong expression in a number of tumor entities [4], [5], [6]. Novel drugs target multiple of these RTKs. Among them is the tyrosine kinase inhibitor sunitinib malate (Sutent) which was recently approved for treatment of gastrointestinal stromal tumors (GIST) and renal cell carcinoma. Sunitinib targets structurally related RTKs including PDGFRα, c-Kit and VEGFR-2. Here we evaluated the effect of sunitinib on MPNST cell lines with characterized 4q12 amplification status, protein and transcript levels. Since gene amplification is a common mechanism to increase expression of oncogenes, we aimed to identify likely target genes of the amplicon. Identification of genes involved in tumor progression and development provides the molecular basis for targeted therapy.

## Materials and Methods

### Tumor tissue, DNA and RNA extraction

Tumor samples were collected from University Hospital Eppendorf (Hamburg, Germany) and Charité – Universitätsmedizin Berlin (Germany). DNA of 10 MPNST from 9 patients (MPNST 21852 and 22318 belong to the same patient), 4 MPNST cell lines (S462, ST88-14, NSF-1, S805), low passage MPNST culture (31002) and dermal fibroblasts was examined. DNA extraction was carried out with QIAamp DNA Mini Kit from Qiagen (Hilden, Germany). With the exception of 31002 cells all other samples originated from NF1 patients. Tumors 22318, 21914, 24472, 24626 and cell line S462 have been analyzed previously by real time PCR and had increased gene dosage of *PDGFRA* and *KIT* [2]. Before extracting nucleic acids and protein lysates a slice of each frozen tumor piece was examined histologically after hematoxylin-eosin staining to exclude contaminating non tumorous portions or necrosis. RNA was extracted using Trizol reagent from Invitrogen (Karlsruhe, Germany) whenever snap frozen samples were available. The quality of all RNAs was examined with a Bioanalyzer (Agilent, Böblingen, Germany). Samples with an RNA integrity number (RIN) below 7 were not included. RIN of cell lines was >9. RNA of nervous tissue and temporal lobe from a patient with pharmacoresistant epilepsy served as control for RT-PCR.

### Ethics Statement

This project was approved by the Charité ethics committee. Investigations were carried out with written consent of the patients.

### MLPA analysis

We designed a set of 36 half probes in order to examine 9 genes on 4q12 and 4 control genes mapping to other regions: 8q12-13 (*IL7*), 3q28 (*FXR1*), 2q35 (*DES*), 4q13 (*EPHA5*). Fourteen probe pairs were designed to bind to genes on the chromosomal segment 4q12. The genes *KIT*, *PDGFRA*, *KDR*, *IGFBP7* and *CHIC2* were each recognized by 2 different probe pairs. A detailed description of the analysis has been described elsewhere [3]. Ratios of >1.5 were scored as gene amplification because lymphocyte DNAs never yielded values higher than 1.3.

### Real time RT-PCR

Elimination of genomic DNA and reverse transcription was achieved with the Quantitect reverse transcription kit (Qiagen). Subsequent PCR reactions were performed with the Quantitect Sybr Green PCR kit in a volume of 12.5µl containing cDNA equivalents of 10–20ng RNA. PCRs were performed in triplicates using the 7900HT Fast Real-time PCR System (Applied Biosystems, Weiterstadt, Germany). Data were accepted as valid if standard deviation of Ct values was <0.5 cycle. Intron spanning primers were designed and melting curves analysis was performed to ensure specific signals. Primer sequences and amplification conditions are given in Table S1. Ct values of the target genes *PDGFRA* (171bp), *KDR* (191bp), *KIT* (180bp), *LNX1* (105bp) and *CHIC2* (209bp) were compared to the reference gene *RPS3* (188bp). *RPS3* was shown to be expressed with similar levels in benign and malignant nerve sheath tumors [2]. PCR efficiency determined by serial dilution of cDNA demonstrated similar results for target and reference genes. ΔCt was defined as Ct (target)−Ct (reference). The value for n-fold amplification of targets in relation to the reference was calculated by n = 2^(ΔCt)^.

### Western blot

Cell cultures were washed with PBS and scraped from the dishes in ice cold PBS. After centrifugation the cell pellet was homogenized in ice cold lysis buffer (1% Triton ×100, 100mM NaCl, 50mM Tris-HCl pH 7.5, 5mM EDTA) containing protease and phosphatase inhibitor cocktail for 30min. After another centrifugation step the supernatants were collected and protein content was measured. Approximately 20µg of the lysates were heat denaturated and loaded on 4–12% gradient gels (Invitrogen) for subsequent protein separation. MagicMark XP from Invitrogen was applied as size standard. After transfer of proteins to nitrocellulose membranes (Invitrogen), the membranes were blocked in 3% non fat dry milk with 0.05% Tween-TBS for 1h and incubated overnight at 4°C with antibodies to PDGFR-β (sc-339), PDGFR-α (sc-338), VEGF (sc-152) and FLK-1 (VEGFR-2, sc-6251) which were diluted 1∶200 and obtained from Santa Cruz Biotechnology, Heidelberg, Germany. The total- and phospho-MAP-Kinase 1/2 antibodies were from Upstate (USA) and diluted 1∶2000. After washing, the membranes were incubated for 1h with a secondary peroxidase labeled secondary antibody. Visualization was performed with advanced ECL (Amersham Biosciences, Freiburg, Germany). To ensure equal loading the membranes were stained with ponceau S (Sigma-Aldrich, Germany) after blotting and rehybridized with antibodies to total MAP-Kinase 1/2 or beta actin (dilution 1∶6.000, AC-15) from Sigma for 2h.

### Cell culture and apoptosis assays

MPNST cell lines, low passage culture 31002 and dermal fibroblasts were maintained in DMEM Glutamax-I (1000mg/L glucose) with 10% FBS and 5µg/mL gentamycin from Invitrogen. *NF1*−/− Schwann cells were cultured as described [7]. Human umbelical vein endothelial cells (HUVECS) were from Lonza und cultured in HUVEC medium. Sunitinib malate (kindly provided by Pfizer Inc) was dissolved in dimethylsulfoxid (DMSO) and 10mM stocks were stored at −80°C. During the drug assays the cells were maintained in DMEM containing 5% FBS. 10^5^ cells were seeded in 300µL medium into 24 well plates and allowed to adhere over night. Sunitinib was added in 100µL to obtain the indicated concentrations. Negative controls contained vehicle only. Cell proliferation was evaluated on day 4 post treatment with the CellTiter 96 AQueous One Solution Cell Proliferation Assay (Promega, Mannheim, Germany). The experiments were performed in triplicates and repeated at least thrice. The standard error of different assays was calculated. The effect of growth factors on downstream signaling was determined in cell culture dishes with a diameter of 10cm. Semiconfluent cells were serum starved for 24h. Cells were then stimulated with 50ng/mL PDGF-AA (Calbiochem, Schwalbach/Ts., Germany), 100ng/mL VEGF-165 (R&D system) or 100ng/mL EGF (Santa Cruz Biotechnology) for 10min at 37°C. To check if sunitinib blocks signaling one dish was incubated with sunitinib for 1h before growth factors were added. Cell lysates were analyzed by western blot.

Apoptosis assay was performed with the Apoptag Peroxidase in situ Oligo Ligation Apoptosis detection kit from Chemicon. Detached cells from the supernatant were collected by using cytospin processing. Cells detached by trypsin treatment served as negative control. The staining procedure was performed according to the instruction of the manufacturer. DAPI I (Vysis, Inc., IL, USA) was utilized to detect apoptotic nuclei.

### Immunocytochemistry

2×10^4^ MPNST cells/well were seeded on Permanox chamberslides (Nunc, Wiesbaden, Germany). Cells were fixed with methanol the following day. The antibodies to VEGFR-2 (dilution 1∶50) and VEGF (dilution 1∶50) from Santa Cruz Biotechnology (see western blot) were incubated for 2h. Visualization was performed with Cy3- (Dianova, Hamburg, Germany) or Alexa488- (Invitrogen) conjugated antibodies (dilution 1∶100). Negative controls without primary antibodies did not produce signals.

### Determination of VEGF expression

VEGF concentration was measured in lysates of cells and tissues (20–30µg/well) with the Multi-array 96-well plate from Meso Scale discovery (Maryland, USA). As positive controls lysates of glioma cell lines were employed. The assay was performed according to the manufacturer's recommendation.

## Results

### Characterization of the chromosomal segment 4q12 in MPNST

**Figure 1:**
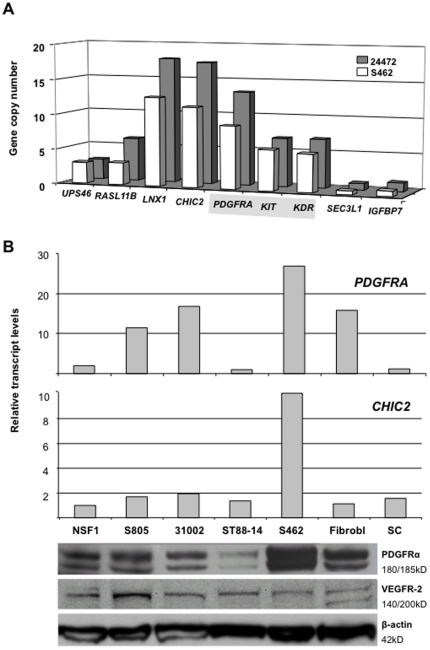
Amplification and expression of genes located on chromosomal segment 4q12. A) Amplification pattern of 9 genes in an MPNST and its corresponding cell line S462. Sunitinib targets are highlighted in grey. B) Transcript and protein levels in 5 MPNST cell lines, dermal fibroblasts and neurofibroma derived Schwann cells (SC).

We analyzed 10 MPNST from 9 patients, 4 MPNST cell lines and low passage culture 31002 by MLPA. Figure 1A depicts the amplification patterns of MPNST 24472 and cell line S462, which was cultivated from this MPNST. The amplicon was maintained at least for 15 passages in the cell line. All samples with altered gene dosage are summarized in Table 1. Cell line ST88-14 was the only sample which had a reduced gene dosage (compatible with allelic loss). MPNST 21852, 22318 and 24626 show increased values in control gene *EPHA5* on chromosomal segment 4q13, 8Mb distal from *IGFBP7*. This observation suggests either chromosome 4 polysomy or an amplicon extending to 4q13.

**Table 1:** Copy numbers of MPNST and MPNST cell lines on chromosomal segment 4q12.

Tumor ID	*UPS46*	*RASL11B*	*LNX1*	*CHIC2*	*PDGFRA*	*KIT*	*KDR*	*SEC3L1*	*IGFBP7*
21852*	2,3	2,4	3,4	2,5	2,7	3,1	2,8	2,6	2,9
22318*	0,9	4,5	2,7	3,9	3,1	2,7	3,1	3,0	3,1
21914	5,6	5,8	5,8	5,0	5,0	5,2	6,0	5,2	6,0
24626	2,1	2,0	1,9	1,6	1,7	1,4	2,1	1,8	1,4
24472#	2,9	6,2	17,8	17,4	13,3	6,9	6,9	0,9	1,2
24772	1,1	1,3	1,1	1,2	1,2	0,9	1,2	1,2	1,0
29250	1,0	0,8	1,0	0,9	1,2	1,0	0,9	0,7	0,8
24484	1,1	1,2	1,1	1,2	1,1	1,1	1,2	1,1	1,1
31472	1,1	1,3	1,3	1,1	1,2	1,3	1,0	1,2	1,1
31474	1,1	1,0	1,4	1,0	1,1	1,2	1,0	1,0	1,1
**S462#**	3,0	3,2	12,7	11,4	8,9	5,8	5,4	0,5	0,8
**ST88-14**	0,5	0,7	0,6	0,6	0,7	0,5	0,7	0,6	0,6
**S805**	1,2	1,2	1,4	1,1	1,3	1,3	1,2	0,7	0,8
**NSF-1**	1,0	0,8	0,9	0,9	1,0	1,2	1,0	0,8	0,9
**31002**	1,0	0,9	1,0	1,1	1,2	1,0	1,3	1,0	1,0
**Fibrobl**	0,8	1,0	1,0	1,1	1,0	0,9	1,0	1,0	1,0

Tumor ID	*EPHA5 (4q13)*	*FXR1 (3q28)*	*DES (2q35)*	*IL7 (8q12-13)*
21852*	2,1	1,3	0,7	3,0
22318*	1,9	1,1	0,8	2,3
21914	0,8	1,2	1,2	4,1
24626	1,7	1,3	1,7	0,6
24472#	1,2	1,2	0,8	1,9
24772	1,1	1,3	1,1	1,2
29250	1,0	0,8	1,0	0,9
24484	1,1	1,0	1,0	1,0
31472	0,6	0,9	0,8	1,4
31474	1,2	1,2	1,1	1,1
**S462#**	0,6	1,1	0,8	1,4
**ST88-14**	0,5	0,9	1,0	0,9
**S805**	0,54	1,1	1,2	1,0
**NSF-1**	0,8	1,0	1,0	1,0
**31002**	1,0	0,9	1,0	1,0
**Fibrobl**	1,1,	1,0	1,0	0,9

Genes are listed in their physical order. Numbers indicate fold increase of gene dosage relative to the normal gene dosage. Tumors retrieved from an identical patient are marked with the same symbol and cell lines and fibroblasts are printed in bold. Control probes on other chromosomal segments than 4q12 are listed in the lower parts of the table.

### Expression levels of genes on chromosomal segment 4q12

**Figure 2:**
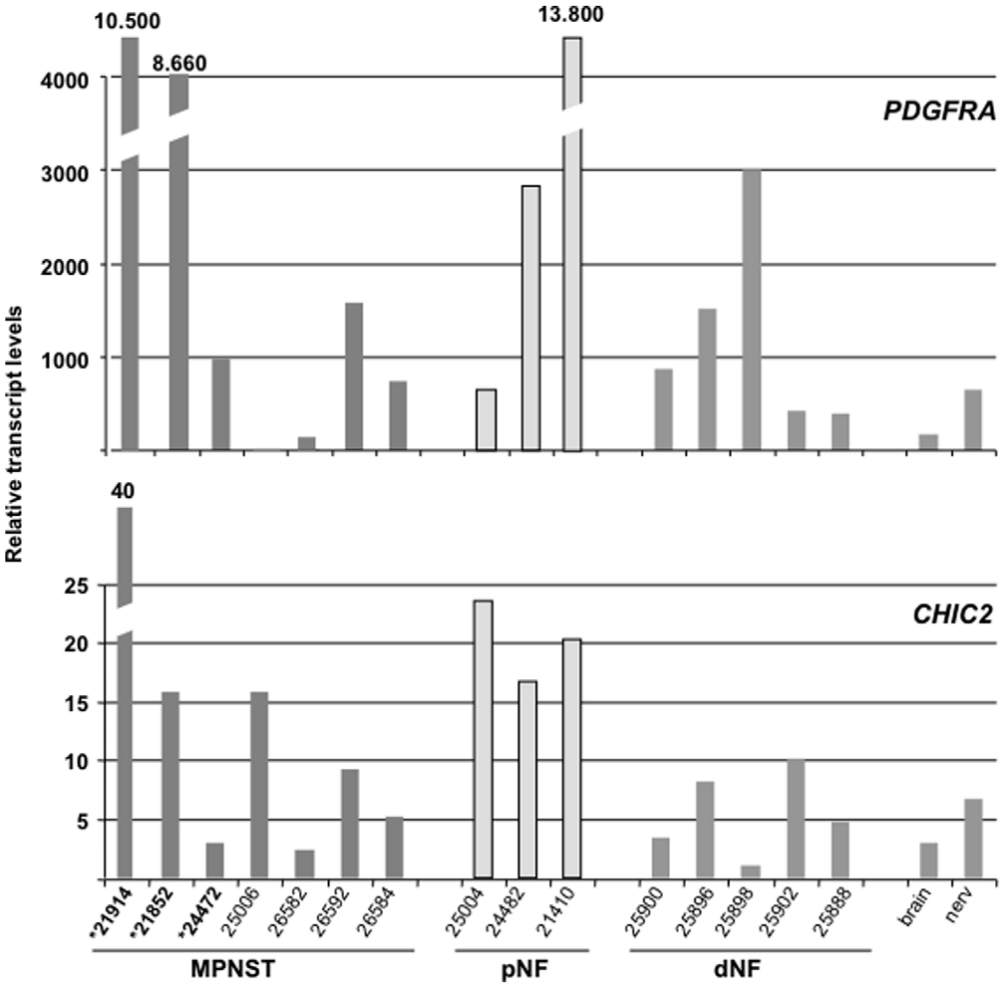
*PDGFRA* and *CHIC2* transcript levels in MPNST, plexiform neurofibromas (pNF) and dermal neurofibromas (dNF) as determined by real time PCR. Normal nervous tissue and brain served as control. PCR was performed in triplicates and accepted when Ct value variation was less than 0.5 cycles. MPNST with gene amplification are marked with *.

To determine consequences of gene amplification and to identify a possible target gene of the amplicon we performed real time RT-PCR. Five MPNST cell lines were analyzed. Human fibroblasts and *NF1*−/− Schwann cells served as controls. We examined expression of the 3 receptor genes *PDGFRA*, *KIT*, and *KDR*, because of their central function in tumor biology and their significance as therapeutic targets. In addition, we determined *CHIC2* and *LNX1* expression because of their high copy numbers (Fig. 1A). *PDGFRA* and *CHIC2* expression correlated significantly with gene dosage found in S462 cells (Fig. 1A and B). Pearson correlation revealed a p-value of 0.05 for *PDGFRA* and <0.01 for *CHIC2*. Correlation coefficients were 0.75 and 0.99, respectively. Expression levels of *KIT* and *LNX1* (Fig. S1) and *KDR* (Fig. 4A), did not correlate with increased copy number found in S462 cells. In a next step we analyzed expression of *PDGFRA* and *CHIC2* in 7 MPNST, 3 pNF and 5 dNF (Fig. 2). MPNST 21914 and 21852 with increased gene dosage, 5.0 and 2.7 respectively, displayed strong *PDGFRA* expression. *CHIC2* expression was particularly strong in MPNST 21914. The data indicate that gene amplification can result in elevated levels of *CHIC2* and *PDGFRA*.

### Protein expression levels of RTKs

Levels of PDGFRα, c-Kit and VEGFR-2 were determined in 5 MPNST cell lines. Weak signals were detected for c-Kit (data not shown) and VEGFR-2 but did not correlate with amplification status of respective cell lines. By contrast, PDGFRα expression showed strong differences between the cell lines (Fig. 1B). Its strong expression in S462 cells matched well with the increased *PDGFRA* copy number in this cell line. Moreover, weak signal in ST88-14 cells correspond to a *PDGFRA* gene dosage of 0.7. *PDGFRA* transcript and protein levels were also in accordance.

### Sunitinib inhibits MPNST cell proliferation, signal transduction and induces apoptosis

**Figure 3:**
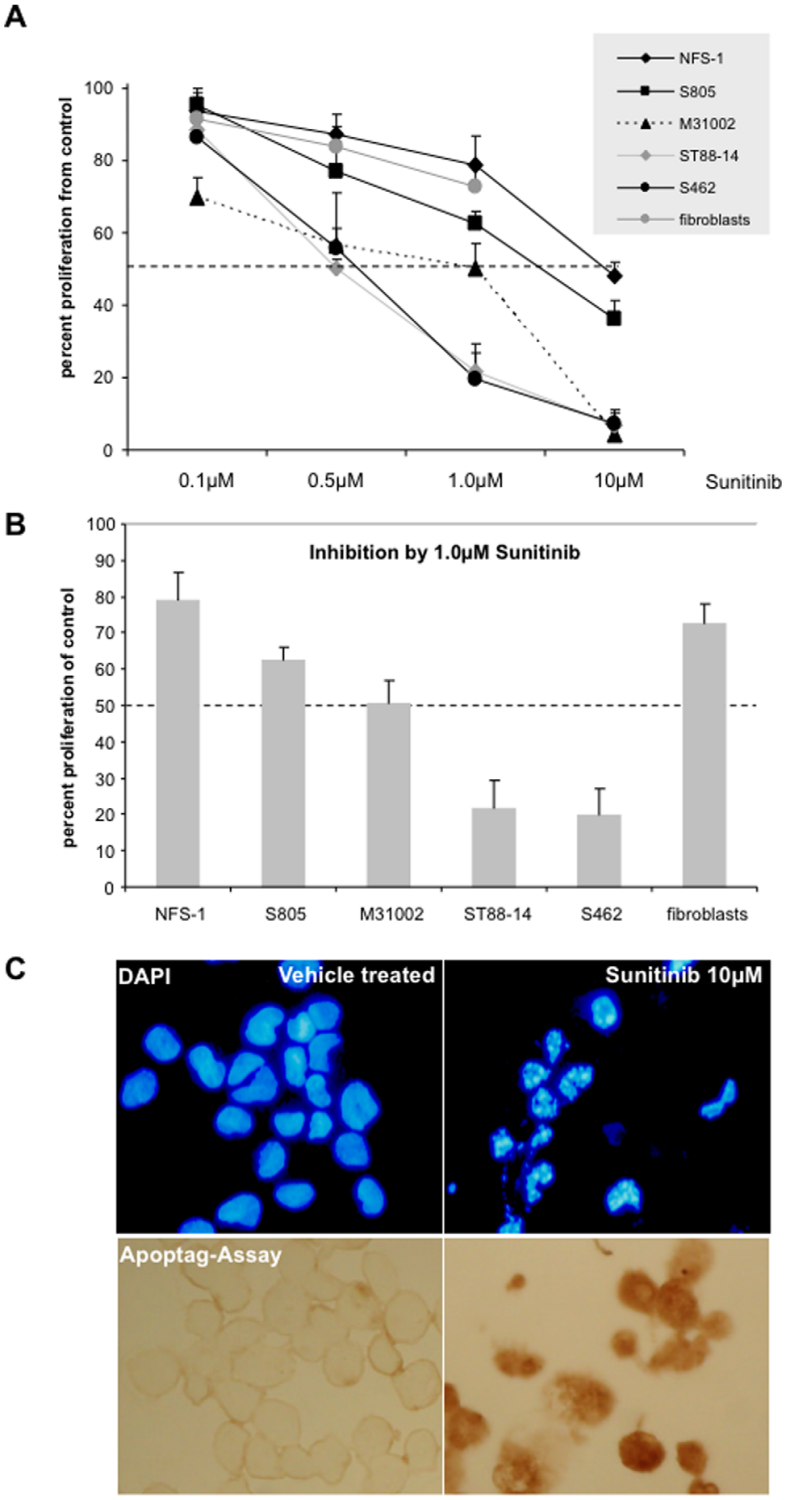
Effect of sunitinib on MPNST cell lines and fibroblasts. A) Dose dependent inhibition of MPNST cell lines after 4 days of treatment. B) For better comparability of cell lines treatment with 1.0 µM sunitinib is depicted in the bar chart. The dashed line marks IC_50_. C) Detection of apoptosis in S462 cells treated for 3 days with 10µM sunitinib. Note formation of apoptotic bodies by DAPI staining (upper panel) and DNA fragmentation as detected by the Apoptag assay (lower panel).

**Figure 4:**
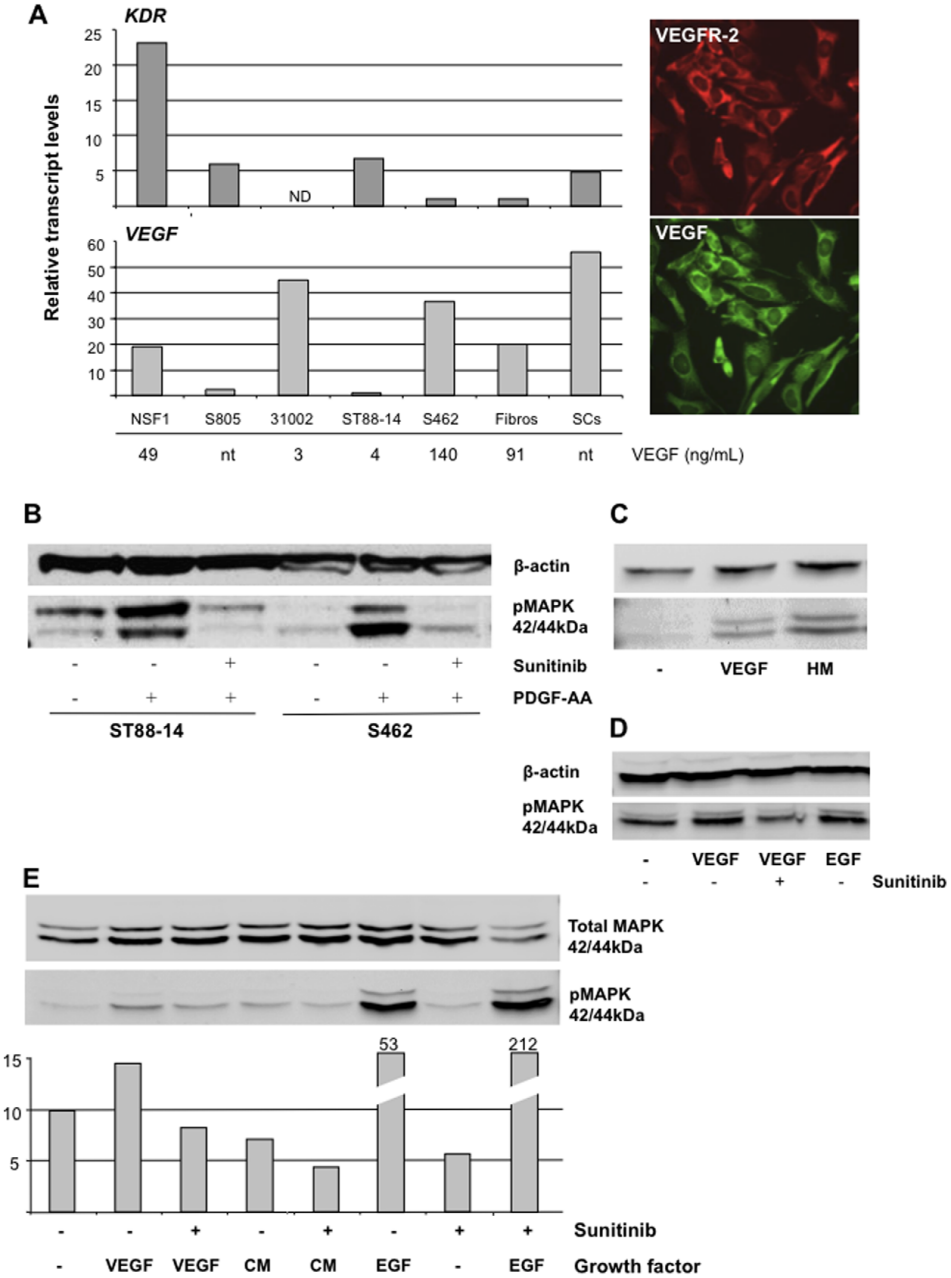
Expression of VEGF and VEGFR-2 in MPNST cell lines and inhibition of growth factor induced signaling by sunitinib. A) Transcript levels of KDR (VEGFR-2) and VEGF. Corresponding concentrations of VEGF in cell lysates is shown below the bar chart (nt: not tested; ND: not detected). On the right side cytochemistry of VEGF and VEGFR-2 in S462 cells is shown. B) Serum starved MPNST cell lines ST88-14 and S462 were stimulated with PDGF-AA (50ng/ml). Where indicated cells were pre-incubated with 5µM sunitinib for 1h to block signaling. C) Serum starved HUVECs were stimulated with VEGF-165 (100ng/ml) or HUVEC medium (HM). D) VEGF-165 (100ng/ml) was used to stimulate serum starved NSF1 cells. Sunitinib blocked VEGF induced signaling. E) VEGF-165 and conditioned S462 medium (CM) was used to stimulate serum starved S462 cells. EGF (100ng/ml) served as positive control. Cells were pre-incubated with 5µM sunitinib where indicated. Densidometric analysis of pMAPK/total MAPK is shown in the bar chart.

The effect of sunitinib was determined on our characterized MPNST cell lines. By testing 4 different concentrations (range 0.1–10µM) we observed a dose dependant inhibition (Fig. 3A B). Cell lines ST88-14 and S462 showed highest sensitivity to the drug. The dose that inhibited cell proliferation by 50% (IC_50_) was 0.5µM for these cell lines. In order to test if sunitinib can induce apoptosis we treated S462 cells for 3 days with 10µM sunitinib. Morphologically, we observed typical signs of apoptosis like cell shrinkage and detachment from culture dishes (data not shown). Detached cells contained blunt end double strand DNA breaks, a typical feature of cells undergoing apoptotis (Fig. 3C). Cells that still adhered to the dish had already formed apoptotic bodies (Fig. 3C). In order to determine the effect of sunitinib on PDGFRα we investigated if the drug would inhibit PDGF-AA induced downstream signaling via PDGFRα. PDGF-AA dimers exclusively bind to PDGFRα homodimers [8]. Thus contributions of other receptors can be excluded. Stimulation with PDGF-AA led to elevated levels of phosphorylated MAP kinase (pMAPK) in both cell lines tested (Fig. 4B). Increase in pMAPK was totally blocked by pre-incubation of 5µM sunitinib.

In a next step we wanted to find out whether the VEGF/VEGFR-2 signaling loop is functional in MPNST cells and might play a role in tumor biology. With different methods we provide evidence for co-expression of VEGF and its receptor (Fig. 1B und 4A). Moreover, we show that VEGF is expressed in solid nerve sheath tumors (Table 2). In order to test if VEGF triggers signal transduction we stimulated MPNST cells with VEGF-165 (100ng/ml). HUVECs served as positive control and showed proper induction of pMAPK upon stimulation with either VEGF or HUVEC medium (Fig. 4C).

**Table 2:** VEGF (ng/mL) expression in solid nerve sheath tumors.

sample	MPNST	pNF	dNF
1	1150 (±13)	14 (±1.6)	3 (±1.6)
2	74 (±11)	3 (±0.8)	8 (±1.2)
3	26 (±0.8)	42 (±0)	
4	19 (±1.2)	5 (±3.6)	
5	15 (±3.7)		

Values given represent the mean of duplicates.

The effect of VEGF was then tested on MPNST cell lines S462 and NSF1. EGF (100ng/ml) was used as positive control. EGF is an important growth factor for MPNST and induced a strong pMAPK signal (Fig. 4D & E). Although less pronounced, addition of VEGF also led reproducibly to elevated pMAPK in S462 and NSF1 cells (Fig. 4D & E). Pre-incubation with sunitinib blocked VEGF induced activation completely in both cell lines. Because growth factors like PDGF and VEGF are produced by MPNST cells we tested serum-free DMEM cultivated for 48h with sub-confluent S462 cells for its ability to induce pMAPK. Conditioned medium (CM) failed to pMAPK increase, which is likely to be explained by low growth factor concentrations.

## Discussion

We show here that multiple genes on chromosomal segment 4q12 are amplified in a subset of MPNST and one MPNST cell line. Taking into account previously analyzed tumors [2] the amplicon is present in about 15% of MPNST and encompasses numerous genes. Previous studies on chromosomal imbalances found chromosomal gains more frequently than losses [9], [10]. This finding supports the idea that oncogenes are more frequently affected in MPNST than tumor suppressor genes, at least in the later stage of the disease. Moreover, both studies found gains of chromosomal arm 4q in MPNST.

Although gene alterations typical for a certain entity are often overrepresented in cell lines as compared to solid tumors they can also be lost during in vitro expansion. This phenomenon has been consistently reported for glioblastoma, which frequently carry amplified *EGFR*. Pandita et al. studied in vitro and in vivo fate of amplified *EGFR* in glioma cells. They could show that the in vivo environment selected for *EGFR* amplification, whereas in vitro expansion selected against this alteration [11]. Here we show that the amplification pattern in cell line S462 reflects the pattern of the corresponding MPNST. Thus, the tumor bulk and the cell population growing in culture are alike concerning the 4q12 amplicon. Cell line S462 appears therefore as a good model, for example for testing drugs that target proteins encoded by genes within the amplicon.

Concerning novel therapeutic approaches the most attractive genes within the amplicon are the 3 structurally related RTKs. In our previous study we showed that PDGFRα is expressed in 75% of MPNST and in MPNST cell lines [2]. This result suggests that *PDGFRA* is an important player in MPNST biology and may be the target gene of the amplicon. By contrast, c-Kit expression, which was also analyzed, showed infrequent and weak expression and was not present in cases that revealed to harbor increased *KIT* copy numbers. These results argue against a prominent role of *KIT* within the amplicon. PDGFRα, a well known oncogene, displays increased copy numbers in different tumor entities including glioblastoma [3], [12]. Glioblastoma and MPNST originate both from a neuroectodermal cell type. Thus, one may speculate that tumorigenesis follows a similar molecular pathogenesis. PDGF is a strong mitogenic factor for glia cells and thought to play a key role in astrocytoma genesis [13]. Since the PDGF/PDGFR system is of major importance in formation of glial tumors it is very likely that gene amplification contributes to increased expression of *PDGFRA*. However, we cannot exclude that other genes within the amplicon contribute to tumor formation or progression. At least *CHIC2* expression was upregulated in some of the samples with underlying gene amplification. Notably, cases of acute myeloid leukemia with a *CHIC2-ETV6* fusion gene have been reported indicating a possible role of *CHIC2* in cancer [14]. However, its function remains largely unknown.

Self-sufficiency in growth signals is among the hallmarks of cancer [15]. Multiple mechanisms can lead to a higher grade of independence from exogenous growth stimuli, for example, increased expression of receptors and ligands or mutated constitutive active receptors. In the context of NF1 such mechanisms are likely to be extra-ordinary effective because prolonged signal transduction takes place in *NF1* deficient cells [16]. Moreover, *NF1* deficient cells express elevated levels of tumor supporting growth factors [17], [18]. Interference with growth factor signaling appears thus attractive in NF1, in particular when the tumors also express high levels of respective receptors. Previously, we have shown that imatinib inhibits proliferation of MPNST cells [2]. Sunitinib and imatinib have an overlapping range of target molecules (PDGFRα, PDGFRβ, c-Kit). However, sunitinib has an even broader spectrum of structurally related receptors including VEGFR1–VEGFR3, and fms-related tyrosine kinase 3 (FLT3) and colony-stimulating factor-1 receptor (CSF-1R) [19], [20], [21]. By targeting a multitude of receptors sunitinib affects different pathways, which mediate angiogenesis, proliferation and invasion of tumor cells. While 10µM of imatinib was necessary to reduce proliferation of S462 cells by 50% only 0.5µM of sunitinib was needed. One reason for more effective inhibition by sunitinib might be its broader target spectrum. Most sensitive to sunitinib were cell lines S462 and ST88-14 although the latter one expressed only little amounts of PDGFRα. However, western blot analysis showed that ST88-14 cells express high levels of the sunitinib target PDGFRβ (data not shown), which may explain the sensitivity of this cell line. Moreover, ST88-14 cells harbor wild type *TP53* making them more sensitive in general. In contrast, S462 cells carry mutant *TP53* [22]. It is well known that the *TP53* status can modulate the effect of receptor targeting drugs [23].

Up to now no clinical studies on sunitinib are available for MPNST patients. However, few MPNST patients have been treated with imatinib [24]. The clinical response was not convincing with only one patient showing stable disease and 4 patients with progressive disease. Mutation or expression data of imatinib targets were not available. We assume that imatinib as single treatment may not be sufficient for most MPNST, because these tumors accumulate multiple alterations during the course of progression. However, molecular analysis of MPNST prior to treatment may help to identify subgroups of patients, which may profit from imatinib or suntinib treatment. Moreover, the combination with other drugs could also improve clinical response. Inhibition of the tumor promoting effect of mast cells mediated by imatinib may be sufficient for pNF [25] but not enough for MPNST.

Besides being a key regulator of angiogenesis VEGF acts also as neuroprotective factor. Protective and mitogenic effects have been reported for neuronal and glial cells in vitro and in vivo [26]. Here we show that VEGF and VEGFR-2 are co-expressed in MPNST cells and that VEGF triggers signal transduction suggesting a functional significance. In an ovarian carcinoma model the VEGF/VEGFR-2 loop protected tumor cells from anoikis [27]. Another study showed a pro-mitogenic autocrine loop in glioblastoma cells which protected from ionizing radiation [28]. Thus, in addition to its pro-angiogenic potential VEGF may have supportive and/or protective effects on tumor cells. Reagents blocking the VEGF/VEGFR-2 loop could thus inhibit tumor growth by two mechanisms: blockage of vessel formation and interference with autocrine signalling.

It is well known that peripheral nerve sheath tumors are highly vascular and can stimulate angiogenesis in vivo [29]. Moreover, tumor derived human Schwann cells from NF1 patients can stimulate angiogenesis and there is evidence for enhanced expression of angiogenic factors in NF1 deficient cells [17] A recent study showed VEGF expression in all MPNST analyzed (n = 22). VEGF expression and micro vessel density was significantly higher in MPNST than in neurofibromas and schwannomas [30] suggesting a role in tumor progression. Our results confirm higher expression of VEGF in MPNST than in neurofibroma (table 2) and provide first evidence for a functional VEGF/VEGFR-2 loop in MPNST. However, further analysis is needed to assess the exact role of VEGF in nerve sheath tumor biology.

Taken together, our data support the idea that sunitinib may be used for treatment of MPNST patients. We show that the drug is effective on MPNST cell lines within the low micro molar range, a concentration that is achieved in patients plasma [31] (An AUC at steady state of 1µg*h/ml corresponds to 1.9µM sunitinib). Because sunitinib targets multiple receptors expressed or over-expressed in MPNST the drug undermines several “survival strategies” of the tumor. Finally, presence of the 4q12 amplicon might serve as a predictive marker for sunitinib response.

## Supporting Information

Figure S1 Transcript expression levels of KIT and LNX1
(3.00 MB TIF)

Table S1 (0.03 MB DOC)
