# Supplementary figures and images for: The 4q12 Amplicon in Malignant Peripheral Nerve Sheath Tumors: Consequences on Gene Expression and Implications for Sunitinib Treatment
Source: PLoS One. 2010 Jul 29;5(7):e11858. doi: 10.1371/journal.pone.0011858 (PMC2912277; doi:10.1371/journal.pone.0011858)

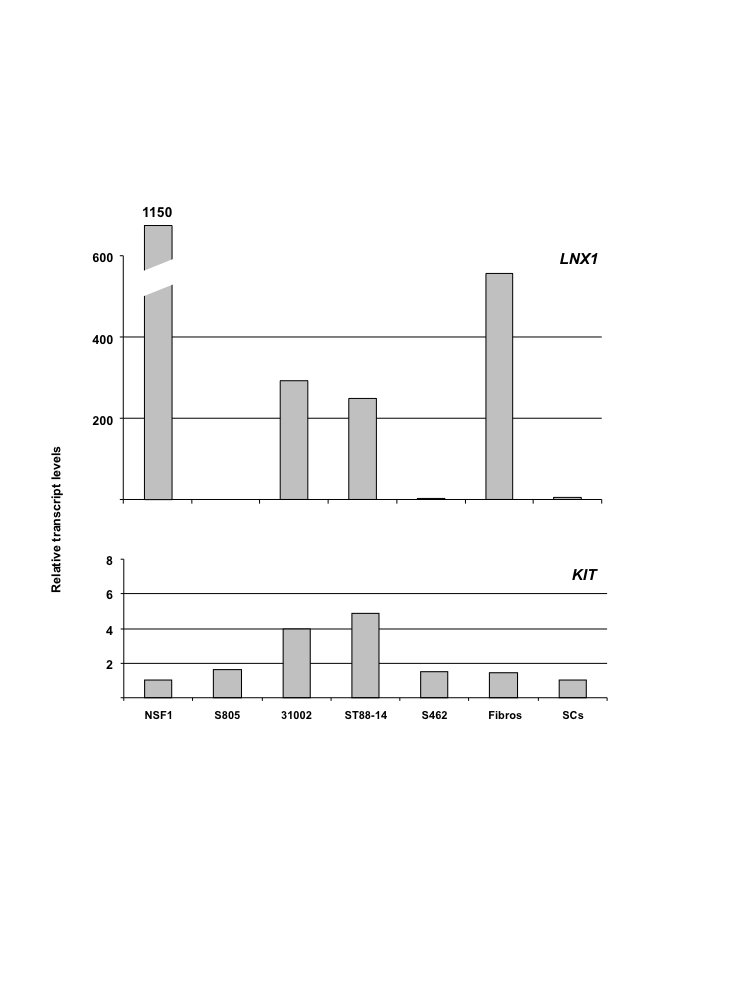

Supplement: Figure S1 — Transcript expression levels of KIT and LNX1 (3.00 MB TIF) [file pone.0011858.s001.tif]
