# Supplementary material for: The 4q12 Amplicon in Malignant Peripheral Nerve Sheath Tumors: Consequences on Gene Expression and Implications for Sunitinib Treatment
Source: PLoS One. 2010 Jul 29;5(7):e11858. doi: 10.1371/journal.pone.0011858 (PMC2912277; doi:10.1371/journal.pone.0011858)
Supplement: Table S1 — (0.03 MB DOC) [file pone.0011858.s002.doc]

# Supplementary Table S1

| Gene name | Primer sequence (5´ - 3´) |
| --- | --- |
| PDGFRA-F | CACCTGAGTGAGATTGTGGA |
| PDGFRA-R | CTTCAGCTTGTCTTCCTCGT |
| KDR-F | TGGTCACCATCTCAATGTGG |
| KDR-R | GGATTGCTCCAACGTAGTCT |
| KIT-F | AAGTGACGTCTGGTCCTATG |
| KIT-R | ATCTGCATCCCAGCAAGTCT |
| LNX1-F | GGAGTCATAAGCAGAGATGG |
| LNX1-R | CAATAATGCCACTGCCTCAC |
| CHIC2-F | GAGCAGCTGCTCAAGTACTC |
| CHIC2-R | CCACAAAGTAGCCAACGTAC |
| RPS3-F | GATGGCAGTGCAAATATCCAA |
| RPS3-R | CCTTCTCACCAAGAACATTCT |

Forward primers are marked with F, reverse primer with R.

The following PCR program was applicable for all primer pairs: 2 min 50°C, 15 min at 94°C followed by 40 cycles of 15sec at 94°C, 30sec at 56°C, and 30sec at 72°C.
